# A genetic polymorphism in the *CAV1 *gene associates with the development of bronchiolitis obliterans syndrome after lung transplantation

**DOI:** 10.1186/1755-1536-4-24

**Published:** 2011-11-01

**Authors:** Elisabeth A Kastelijn, Coline HM van Moorsel, Karin M Kazemier, Suzan M Roothaan, Henk JT Ruven, Johanna M Kwakkel-van Erp, Ed A van de Graaf, Pieter Zanen, Diana A van Kessel, Jan C Grutters

**Affiliations:** 1 Centre of Interstitial Lung Diseases, Department of Pulmonology, St Antonius Hospital, Postbox 2500, NL-3420 EM, Nieuwegein, The Netherlands; 2 Division Heart and Lungs, University Medical Centre Utrecht, Postbox 85500, NL-3508 GA, Utrecht, The Netherlands; 3 Department of Pathology, University Medical Centre Utrecht, Postbox 85500, NL-3508 GA, Utrecht, The Netherlands; 4 Department of Clinical Chemistry, St Antonius Hospital, Postbox 2500, NL-3420 EM, Nieuwegein, The Netherlands

**Keywords:** caveolin 1, genetic polymorphism, serum, lung transplantation, bronchiolitis obliterans syndrome

## Abstract

**Background:**

Caveolin 1 (Cav-1) is the primary structural component of cell membrane invaginations called 'caveolae'. Expression of Cav-1 is implicated in the pathogenesis of pulmonary fibrosis. Genetic polymorphisms in the *CAV1 *gene influence the function of Cav-1 in malignancies and associate with renal allograft fibrosis. Chronic allograft rejection after lung transplantation, called 'bronchiolitis obliterans syndrome' (BOS), is also characterised by the development of fibrosis.

In this study, we investigated whether *CAV1 *genotypes associate with BOS and whether Cav-1 serum levels are influenced by the *CAV1 *genotype and can be used as a biomarker to predict the development of BOS.

**Methods:**

Twenty lung transplant recipients with BOS (BOS^pos^), ninety without BOS (BOS^neg^) and four hundred twenty-two healthy individuals donated DNA samples. Four SNPs in *CAV1 *were genotyped. Serial Cav-1 serum levels were measured in a matched cohort of 10 BOS^pos ^patients and 10 BOS^neg ^patients. Furthermore, single-time point Cav-1 serum levels were measured in 33 unmatched BOS^neg ^patients and 60 healthy controls.

**Results:**

Homozygosity of the minor allele of *rs3807989 *was associated with an increased risk for BOS (odds ratio: 6.13; *P *= 0.0013). The median Cav-1 serum level was significantly higher in the BOS^pos ^patients than in the matched BOS^neg ^patients (*P *= 0.026). Longitudinal analysis did not show changes in Cav-1 serum levels over time in both groups. The median Cav-1 serum level in the group of 43 BOS^neg ^patients was lower than that in the healthy control group (*P *= 0.046).

In lung transplant recipients, homozygosity of the minor allele of *rs3807989 *and *rs3807994 *was associated with increased Cav-1 serum levels.

**Conclusion:**

In lung transplant recipients, the *CAV1 *SNP *rs3807989 *was associated with the development of BOS and Cav-1 serum levels were influenced by the *CAV1 *genotype.

## Background

Caveolae are 50- to 100-nm flask-shaped cell membrane invaginations in which the primary structural component is caveolin 1 (Cav-1) [1]. Cav-1 has been found in many cell types, but is abundantly expressed in endothelial cells, type 1 pneumocytes, epithelial cells, smooth muscle cells and fibroblasts [2-5]. It has many cellular functions, including vesicular transport, signal transduction and cholesterol homeostasis [1,4,6].

Kasper *et al. *[5] were the first investigators to link Cav-1 to a fibrotic phenotype in the lungs of rats. Subsequently, studies of the role of Cav-1 in pulmonary fibrosis in humans were conducted. In patients with idiopathic pulmonary fibrosis (IPF), Cav-1 mRNA expression was found to be reduced in epithelial cells and fibroblasts [7]. In patients with systemic sclerosis, Cav-1 expression was markedly decreased in tissue of affected lungs and skin [8]. Knockdown of Cav-1 resulted in a fivefold increase of collagen gene expression by normal human lung fibroblasts, whereas increased Cav-1 expression caused a reduction in collagen [9]. *CAV1*^-/- ^mice developed pulmonary and skin fibrosis [8]. On the other hand, during fibrogenesis, increased expression of Cav-1 was observed in endothelial cells [5,10]. Taken together, the results of these studies support a pivotal role for Cav-1 in the fibrogenesis of the lungs [8,11].

The *CAV1 *gene is localised on chromosome 7, a highly conserved region that includes a known fragile site which is deleted or associated with loss of heterozygosity in a variety of human cancers [12]. Studies that have addressed whether genetic variations of *CAV1 *increase propensity towards fibrosis are scarce. Among kidney transplant donors, a SNP in *CAV1 *was significantly associated with renal allograft fibrosis in two independent cohorts [13].

After lung transplantation, the major limitation on long-term survival is the development of chronic rejection in the form of obliterative bronchiolitis (OB) or its clinical surrogate marker, the bronchiolitis obliterans syndrome (BOS) [14]. OB is characterised by inflammation and remodelling of the pulmonary epithelium of the small airways [15]. This process results in the recruitment and proliferation of fibroblasts, which ultimately leads to fibrosis. Advanced OB can include a spectrum ranging from partial to complete acellular fibrotic obliteration whereby only scar tissue remains of the airway lumen [16,17]. When BOS is diagnosed on the basis of a decline in lung function, the process of inflammation and fibrosis is usually at an advanced and irreversible stage and treatment options are limited [15]. This emphasises the need for biomarkers that predict the development of BOS before a decline in lung function has occurred.

The primary objective of this study was to determine whether SNPs in the *CAV1 *gene are associated with the development of BOS after lung transplantation. In addition, Cav-1 serum levels in controls and lung transplant recipients were measured to evaluate whether Cav-1 serum levels are influenced by genotype and can be useful as a biomarker to predict the development of BOS. To establish whether Cav-1 expression is indeed present in OB lesions, lung tissue sections from BOS^pos ^patients and controls were studied using immunohistochemical staining.

The role of Cav-1 in lung transplant recipients and BOS has never been investigated. However, the role of Cav-1 in another pulmonary fibrotic disease, such as IPF, has been described previously [7]. To improve the understanding of Cav-1, we also measured Cav-1 serum levels in patients with IPF.

## Results

### Genotype and haplotype distribution of CAV1 in patients and controls

During the study period, 139 lung transplant procedures were performed in 138 patients. One hundred ten recipients gave their written informed consent and donated DNA, of whom twenty patients developed BOS during follow-up (Table 1). The genotype distributions of the four SNPs in *CAV1 *in the different groups are reported in Table 2. All SNPs were found to be polymorphic and in Hardy-Weinberg equilibrium (HWE). The genotype distribution of *rs3807989 *was significantly different between BOS^pos ^and BOS^neg ^patients (*P *= 0.015), which is related to a significantly higher minor allele frequency of *rs3807989 *in BOS^pos ^patients than in BOS^neg ^patients (minor allele frequency 0.58 vs 0.35; *P *= 0.027). Homozygotes of this minor allele had an increased risk of developing BOS compared with carriers of the major allele (odds ratio, 6.13; *P *= 0.0013; 95% confidence interval, 1.85 to 20.41). For the other SNPs, no significant differences were found in the genotype distribution and allele frequency between the patient groups and healthy controls.

**Table 1:** Baseline characteristics of BOS^pos ^and BOS^neg ^patients and healthy controls^a^

Variables	BOS^pos^	BOS^neg^	Controls
Number of patients	20	90	422
Gender, *n *(%)			
Male	9 (45%)	46 (51%)	228 (54%)
Female	11 (55%)	44 (49%)	194 (46%)
Mean age (± SD), years	53.4 ± 10.7	49.4 ± 12.7	48.2 ± 11.9
Diagnosis, *n *(%)			NA
COPD	7 (35%)	29 (32%)	
CF	2 (10%)	24 (27%)	
IPF	4 (20%)	13 (14%)	
Sarcoidosis	2 (10%)	3 (3%)	
α1 antitrypsin deficiency	5 (25%)	7 (8%)	
Other	0	14 (16%)	
Type of graft, *n *(%)			NA
Unilateral	4 (20%)	13 (14%)	
Bilateral	16 (80%)	77 (86%)	
Mean time to BOS (± SD), months	23.7 ± 15.2	NA	NA

^a^BOS^pos^, bronchiolitis obliterans syndrome-positive; BOS^neg^, bronchiolitis obliterans syndrome-negative; COPD, chronic obstructive pulmonary disease; CF, cystic fibrosis; IPF, idiopathic pulmonary fibrosis; NA, not applicable; SD, standard deviation.

**Table 2:** Genotype distribution of BOS^pos ^and BOS^neg ^patients and healthy controls^a^

			BOS^pos ^(*n *= 20)			BOS^neg ^(*n *= 87)^b^			Controls (*n *= 422)		
SNPs	Gene region	Major/minor	AA	AB	BB	AA	AB	BB	AA	AB	BB
*rs12154695*	Unknown	C/A	12 (60)	6 (30)	2 (10)	34 (39)	47 (54)	6 (7)	181 (43)	189 (45)	52 (12)
*rs10256914*	Intron	T/C	12 (60)	6 (30)	2 (10)	50 (57)	29 (33)	8 (9)	228 (54)	158 (37)	36 (9)
*rs3807989*^c^	Intron	C/T	4 (20)	9 (45)	7 (35)	33 (38)	47 (54)	7 (8)	144 (34)	206 (49)	72 (17)
*rs3807994*	Intron	C/T	10 (50)	7 (35)	3 (15)	57 (66)	28 (32)	2 (2)	240 (57)	156 (37)	26 (6)

^a^All data are *n *(%). BOS^pos^, bronchiolitis obliterans syndrome-positive; BOS^neg^, bronchiolitis obliterans syndrome-negative; A, major; B, minor. ^b^*CAV1 *genotyping failed in three BOS^neg ^patients. ^c^Genotype distribution BOS^pos ^vs BOS^neg^: *P *= 0.015; allele frequency BOS^pos ^vs BOS^neg^: *P *= 0.027.

The linkage disequilibrium (LD) structure revealed one haplotype block between *rs3807989 *and *rs3807994 *with D' = 1 and *r*^2 ^= 0.45; therefore, haplotypes of the *CAV1 *polymorphisms were constructed and analysed. Fourteen haplotypes were constructed, and the six most frequent haplotypes with a frequency exceeding 5% were used for further analysis (Table 3). BOS^pos ^patients had significantly more homozygotes of haplotype 3 than BOS^neg ^patients and controls (*P *= 0.03).

**Table 3:** Haplotype distribution in BOS^pos ^patients (*n *= 20), BOS^neg ^patients (*n *= 87) and healthy controls (*n *= 422)^a^

Haplotypes	Homozygotes, *n *(%)	Heterozygotes, *n *(%)	Carriers, *n *(%)
CTCC			
BOS^pos^	1 (5)	9 (45)	10 (50)
BOS^neg^	12 (14)	49 (56)	61 (70)
Controls	61 (14)	218 (52)	279 (66)
CTTC			
BOS^pos^	1 (5)	4 (20)	5 (25)
BOS^neg^	0 (0)	12 (14)	12 (14)
Controls	2 (0.5)	52 (12)	54 (13)
CCTT^b^			
BOS^pos^	2 (10)	3 (15)	5 (25)
BOS^neg^	0 (0)	15 (17)	15 (17)
Controls	4 (1)	75 (18)	79 (19)
ATCC			
BOS^pos^	0 (0)	4 (20)	4 (20)
BOS^neg^	0 (0)	20 (23)	20 (23)
Controls	10 (2)	60 (14)	70 (16)
ATTC			
BOS^pos^	0 (0)	4 (20)	4 (20)
BOS^neg^	0 (0)	17 (20)	17 (20)
Controls	4 (1)	76 (18)	80 (19)
ACTT			
BOS^pos^	0 (0)	1 (5)	1 (5)
BOS^neg^	0 (0)	10 (11)	10 (11)
Controls	1 (0.5)	70 (17)	71 (17)

^a^BOS^pos^, bronchiolitis obliterans syndrome-positive; BOS^neg^, bronchiolitis obliterans syndrome-negative; ^b^BOS^pos ^vs controls: *P *= 0.03; BOS^pos ^vs BOS^neg^: *P *= 0.03. Sequence of SNPs in haplotype: *rs12154695*, *rs10256914*, *rs3807989*, *rs3807994*.

### Cav-1 serum levels in patients and controls

In our cohort of lung transplant recipients, serum samples were collected from 10 BOS^pos ^patients and 43 BOS^neg ^patients (Table 4). Initially, we matched 10 BOS^pos ^patients with 10 BOS^neg ^patients to reduce the influence of confounding factors and performed longitudinal analysis of Cav-1 serum levels. These patients were matched for several clinicodemographic variables, including age, gender and primary lung pathology. Serial serum samples were used to perform this longitudinal analysis, and two to five serum samples were collected for every matched BOS^pos ^patient and BOS^neg ^patient.

**Table 4:** Baseline characteristics of BOS^pos ^and BOS^neg ^patients (BOS^neg ^matched and unmatched) and healthy controls and patients with idiopathic pulmonary fibrosis^a^

Variables	BOS^pos^	BOS^neg ^(matched)	BOS^neg ^(unmatched)	Controls	IPF
Total number	10	10	33	60	25
Gender, *n*					
Male	3	4	18	30	18
Female	7	6	15	30	7
Mean age (± SD), years	45.2 ± 15.0	45.7 ± 13.1	48.2 ± 13.9	46.7 ± 11.3	64.7 ± 11.3
Diagnosis, *n*				NA	NA
COPD	3	3	8		
CF	4	5	10		
IPF	1	0	5		
Sarcoidosis	1	0	2		
α1 antitrypsin deficiency	1	1	3		
Other	0	1	5		
Type of graft, *n*				NA	NA
Bilateral	10	10	27		
Unilateral	0	0	6		
Survival, mean ± SD, months	33.6 ± 20.0	46.4 ± 9.5	51.6 ± 21.3	NA	NA
Mean BOS-free survival (± SD), months	19.3 ± 12.5	46.4 ± 9.5*	51.6 ± 21.3*	NA	NA
BOS grade at diagnosis, *n*		NA	NA	NA	NA
1	7				
2	3				
3	0				
Histology		NA	NA	NA	NA
Biopsy, histological OB	4				
Biopsy, no histological OB	2				
No biopsy	4				

^a^BOS^pos^, bronchiolitis obliterans syndrome-positive; BOS^neg^, bronchiolitis obliterans syndrome-negative; * identical to survival; COPD, chronic obstructive pulmonary disease; CF, cystic fibrosis; IPF, idiopathic pulmonary fibrosis; OB, obliterative bronchiolitis; NA, not applicable; SD, standard deviation.

**Figure 1:**
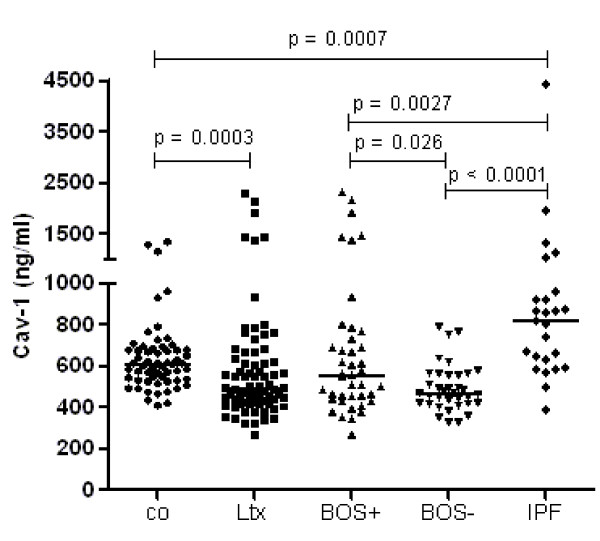
**Caveolin 1 (Cav-1) serum levels in patient groups and healthy controls**. Groups: Controls (co, *n *= 60), the cohort of matched lung transplant recipients (Ltx) (*n *= 10 BOS^pos ^patients and *n *= 10 matched BOS^neg ^patients; samples per patient ranged from two to five), BOS^pos ^patients (BOS+, *n *= 10), matched BOS^neg ^patients (BOS-, *n *= 10), patients with idiopathic pulmonary fibrosis (IPF, *n *= 25). Horizontal bars represent medians.

The median (interquartile range (IQR)) Cav-1 serum level of all samples in the 10 BOS^pos ^patients was significantly higher than that of the 10 matched BOS^neg ^patients: 555 ng/mL (447 to 747) and 468 ng/mL (418 to 558), respectively (*P *= 0.026) (Figure 1). The median Cav-1 serum level of healthy controls (*n *= 60; one sample per individual) was 609 ng/mL (531 to 678) and differed significantly from that of all samples from the 20 matched lung transplant recipients (10 BOS^pos ^and 10 BOS^neg ^patients): 492 ng/mL (426 to 629) (*P *= 0.0003) (Figure 1).

We analysed Cav-1 serum levels in the matched cohort from the time of lung transplantation until the BOS diagnosis was made. Samples were selected using a quadrant-based sampling model. In this model, the follow-up period after lung transplantation until the development of BOS was divided into four equal quadrants, and one sample taken at the midpoint of each interval was analysed. The samples from the BOS^neg ^patients were obtained from chronologically similar visits at which the samples for their BOS^pos ^counterparts were analysed. In the BOS^pos ^patients, one extra sample was obtained within two months before the BOS diagnosis was made. The mean time period between lung transplantation and the onset of BOS was 19 months, with a variation ranging from 8 to 49 months (Table 4). The serial Cav-1 serum levels in both groups did not reveal a significant increase or decrease at similar time points after lung transplantation and prior to BOS. Because Cav-1 serum levels did not change over time, the samples from the first quadrant of this matched cohort were used for further analysis.

To investigate whether the difference in Cav-1 serum levels between the matched BOS^pos ^and BOS^neg ^patients would hold in an unmatched BOS^neg ^group, 33 extra unmatched BOS^neg ^patients were added to the cohort of 10 BOS^neg ^patients. In these 33 BOS^neg ^patients, Cav-1 serum levels were measured at one moment after lung transplantation. We found that the median Cav-1 serum levels were not significantly different between the 43 BOS^neg ^patients and 10 BOS^pos ^patients, respectively: 550 ng/mL (433 to 736) and 565 ng/mL (421 to 738), respectively (*P *= 0.89). The 43 BOS^neg ^patients had significantly lower Cav-1 serum levels than the controls (*P *= 0.046).

To improve the understanding of the role of Cav-1 in pulmonary fibrosis, Cav-1 serum levels were also measured in patients with IPF (*n *= 25; one sample per individual). The median Cav-1 serum concentration in this group was 818 ng/mL (609 to 940), which was significantly higher than that in 10 BOS^pos ^patients (*P *= 0.0027), 10 BOS^neg ^patients (*P *< 0.0001) and controls (*P *= 0.0007) (Figure 1).

### Correlation of Cav-1 serum levels with genotype and haplotype

**Figure 2:**
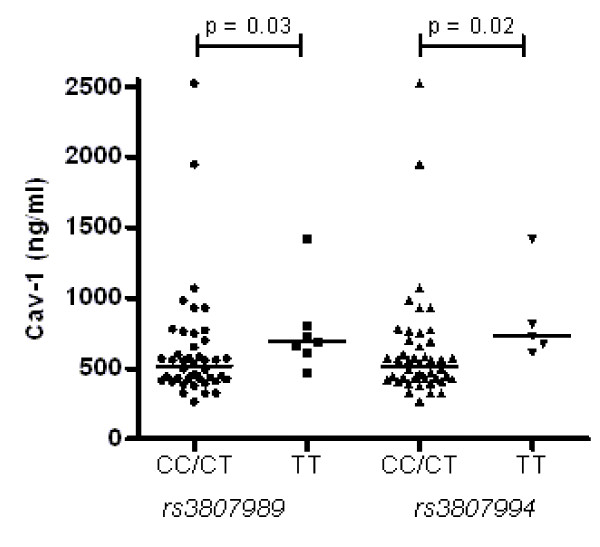
**Correlation of caveolin 1 serum levels with genotype in lung transplant recipients**. Caveolin 1 (Cav-1) serum levels in lung transplant recipients (10 BOS^pos ^and 43 BOS^neg ^patients) are increased in homozygotes of the minor alleles compared with carriers of the major allele of *rs3807989 *(*P *= 0.03) and *rs3807994 *(*P *= 0.02). Horizontal bars represent medians.

In all lung transplant recipients (10 BOS^pos ^and 43 BOS^neg ^patients), homozygotes of the minor allele of the following SNPs had significantly increased serum levels compared with the carriers of the major alleles: *rs3807989 *(689 vs 520 ng/mL; *P *= 0.03) and *rs3807994 *(731 vs 520 ng/mL; *P *= 0.02) (Figure 2).

**Figure 3:**
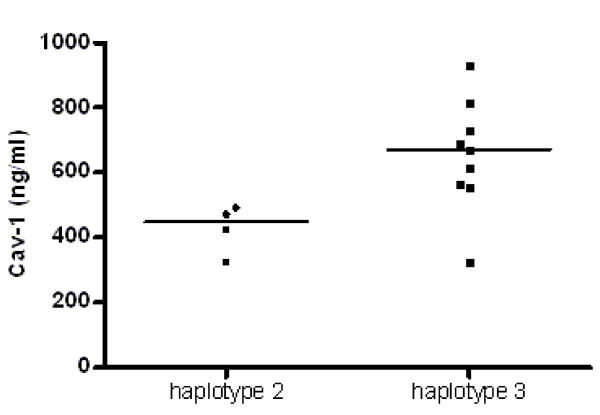
**Caveolin 1 serum levels in carriers of haplotype 2 and haplotype 3 in lung transplant recipients**. Carriers of haplotype 2 (CTTC, *n *= 4) had lower caveolin 1 (Cav-1) serum levels compared with carriers of haplotype 3 (CCTT, *n *= 9) (*P *= 0.02). The number of individuals is smaller than reported in Table 3 because serum samples were not available from all BOS^pos ^and BOS^neg ^patients. Horizontal bars represent medians.

Heterozygotes of haplotype 2 (CTTC) had lower Cav-1 serum levels than heterozygotes of haplotype 3 (CCTT) (448 vs 689 ng/mL; *P *= 0.04). Also, carriers of haplotype 2 had lower Cav-1 serum levels than carriers of haplotype 3 (448 vs 672 ng/mL; *P *= 0.02) (Figure 3).

### Localisation of Cav-1 in obliterative bronchiolitis

**Figure 4:**
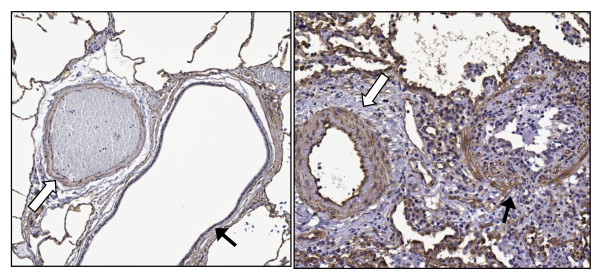
**Immunohistochemical staining for caveolin 1 in lung tissue sections**. Left: Control tissue from a lobectomy specimen (left lower lobe) with positive staining of alveolar epithelium, endothelium and smooth muscle cells and minimal staining of the bronchiolar epithelium. Right: One representative sample of an obliterative bronchiolitis (OB) lesion from the right lower lobe, with some staining of the cellular infiltrate partially obliterating the bronchiole. In this sample, the staining of bronchiolar epithelium seems slightly increased. The staining of alveolar epithelium and endothelium are similar to normal lung tissue. Open arrows indicate the arterial branch. Closed arrows indicate the bronchiole (original magnification, ×100).

Qualitative immunohistochemical staining of Cav-1 was studied in lung tissue from six lung transplant recipients and two controls. In normal lung tissue, the Cav-1 staining was intense in the cell membranes of endothelial cells and the alveolar epithelium. The Cav-1 staining in the bronchiolar epithelial cells was less intense (Figure 4, left panel).

In OB lesions, the intensity of the Cav-1 staining in endothelial cells and alveolar epithelium was similar to that in normal lung tissue. The staining of Cav-1 in the bronchiolar epithelium of lung transplant recipients with OB was slightly accentuated compared with normal bronchiolar epithelium. In the OB lesions, some staining of the cellular infiltrate, that is, partially obliterating the bronchiolus, was observed (Figure 4, right panel).

## Discussion

We found an association between a genetic polymorphism in the *CAV1 *gene and the development of BOS. Homozygosity of the minor allele of *rs3807989 *is associated with a sixfold increased risk of developing BOS. Cav-1 serum levels were genotype-dependent. In lung transplant recipients, increased Cav-1 serum levels were observed in homozygotes of the minor alleles of two SNPs, including *rs3807989*, which genotype was associated with an increased risk of BOS.

Cav-1 is an integral protein of caveolae and has been identified in a wide variety of cells [1]. Although this protein was originally identified as a membrane protein, Cav-1 has also been reported to be present in the secretory cellular components of the pancreas and salivary glands, in differentiating osteoblasts and in cancer cells [18-21]. This might explain the detectable serum levels of Cav-1 in healthy controls. Cav-1 serum levels were never measured before in lung transplant recipients or in patients with IPF. However, Cav-1 serum levels in patients with prostate cancer have been shown to be a potential biomarker in this disease [19,22-24]. We found that Cav-1 in serum was lower in lung transplant recipients than in healthy controls. Within the matched cohort of 10 BOS^pos ^and 10 BOS^neg ^patients, the BOS^pos ^patients had higher Cav-1 serum levels than the BOS^neg ^patients. The six highest Cav-1 serum levels in the BOS^pos ^cohort, illustrated in Figure 1, were measured in two patients who are both homozygous for the risk allele of SNP *rs3807989*. Serial Cav-1 serum levels of the matched cohort did not reveal a trend in Cav-1 concentration with time after lung transplantation and prior to BOS. The total group of 43 BOS^neg ^patients had lower Cav-1 serum levels than the healthy controls, but in contrast to the matched cases there was no difference in Cav-1 serum concentration between the 10 BOS^pos ^patients and the total group of 43 BOS^neg ^patients. For these reasons, Cav-1 serum levels cannot be used as a biomarker to predict the development of BOS.

We also measured Cav-1 serum levels in 25 patients with IPF. Although the pathogenesis of BOS and IPF remains to be determined, there may be some similarities. The general hypothesis is that BOS and IPF are caused by injury of the lung followed by an aberrant repair response and ultimately fibrosis [15,25]. During fibrogenesis in IPF and BOS, the epithelial to mesenchymal transition is a critical cellular mechanism [25,26], and neutrophils play an important role in both diseases [27,28]. Differences between these two entities are the localisation of the lesions in the lung and the stimuli that cause the initial injury. BOS is localised within the respiratory bronchioli and IPF is localised within the alveoli [17,29]. Several immune-dependent and -independent mechanisms are known to be risk factors for the development of BOS [15]. However, the stimuli that cause the injury in IPF are still unknown [25].

Cav-1 serum levels are significantly increased in patients with explicit pulmonary fibrosis, as illustrated by the increased Cav-1 serum levels in patients with IPF in this study. On the other hand, the expression of Cav-1 in lung tissue and fibroblasts of patients with IPF was previously described to be decreased and associated with enhanced transforming growth factor (TGF)-β1 signalling and increased collagen deposition [2,3,7]. The relationship between Cav-1 expression in lung tissue and pulmonary fibroblasts and the Cav-1 serum concentration is unknown. One can only speculate about the discrepancy between the decreased Cav-1 expression in IPF described in the literature and the increased concentration of its soluble form in patients with IPF. Cav-1 is present in caveolae of the cell membrane, and it might also be part of secretory pathways, that is, of the pancreas or salivary glands, which might influence Cav-1 serum levels [18].

Increased Cav-1 serum levels in BOS^pos ^patients compared with BOS^neg ^patients were expected because of the process of fibrogenesis in BOS. In the matched cohort, we were able to detect this difference in Cav-1 concentration. The hypothesis that Cav-1 is increased in BOS^pos ^patients is supported by the correlation of *rs3807989 *minor T allele with both an increased risk of BOS and increased Cav-1 serum levels. For future studies, the presence of extremely high Cav-1 serum levels might be specific to BOS^pos ^patients.

The increased Cav-1 serum levels in BOS^pos ^patients compared with the matched BOS^neg ^patients may be explained by TGF-β1. Researchers in several studies have shown that TGF-β1 is involved in the development of BOS [30-33], although others could not confirm this [26,34].

During pulmonary fibrosis, it has been shown that Cav-1 expression is decreased in epithelial cells and fibroblasts compared with controls, but is increased in endothelial cells [5,7,10,35]. We found that Cav-1 expression in normal lung tissue was similar to the findings of Odajima *et al. *[36], who localised Cav-1 in normal lung tissue and in lung tissue of patients with interstitial pneumonias. A description of Cav-1 expression in OB after lung transplantation is not available. We found that in OB lesions, Cav-1 expression in the bronchiolar epithelial cells seemed to be slightly increased compared with normal lung tissue. In the OB lesions, cellular infiltrates were observed that showed some degree of Cav-1 expression and were partially obliterating the lumen of the bronchiole. These lesions may represent an early phase in the development of BOS and might explain the tendency towards an overall increase in Cav-1 serum levels in BOS^pos ^patients. In the development of BOS, fibrointimal changes involving pulmonary arteries and veins are seen, but they have been overshadowed by the airway lesions [37].

Cav-1 expression in BOS seems increased, but Wang *et al. *[7] found a decreased epithelial expression of Cav-1 in IPF. The limited number of tissue sections from BOS^pos ^patients and the absence of multiple comparisons based on image analysis clearly limit the conclusions we can draw from this part of our study. Pulmonary fibrosis in patients with IPF and systemic sclerosis is different from that in patients with BOS and is localised in other parts of the lung. Different cell types and molecular pathways may be involved in the pathogenesis of these diseases. Furthermore, there is evidence that different types of caveolae exist and that there is more than one regulatory mechanism of Cav-1 expression [35]. Two Cav-1 isoforms, α and β, are known, both of which were detected by the antibodies used in our study [35,38]. The α isoform is mainly expressed by endothelial cells, and the alveolar cells predominantly express the β isoform [38]. This underlines the complexity of Cav-1 in pulmonary pathology.

Regarding the source of the serum Cav-1 in BOS^pos ^patients, we hypothesize that the Cav-1 expression in OB lesions could have a relationship with the increased serum levels we observed. However, further research based on quantitative analysis is needed.

The mechanism by which Cav-1 contributes to fibrosis might be found in the signalling pathway. Cav-1 functions as part of the TGF-β pathway through its participation in TGF-β receptor internalisation. TGF-β is involved in the development of fibrosis by stimulation of extracellular matrix production and accumulation of collagens and other matrix proteins [2,39]. In addition, Cav-1 serves as a scaffolding protein for other signalling molecules, such as members of the mitogen-activated protein kinase family, G proteins and other growth factor receptors [40]. These signalling molecules are involved in the regulation of α-smooth muscle actin-positive fibroblasts and collagen [40,41].

Some limitations of our study have to be acknowledged. First, the study has a retrospective design and the number of patients may be too low to detect more subtle differences in Cav-1 serum levels within the lung transplant recipients. However, the procedure that we used to match the BOS^pos ^and BOS^neg ^patients might have reduced the influence of confounding factors. Additional studies with more lung transplant recipients and a longer follow-up period are required to replicate our association between the *CAV1 *genotype and BOS. In addition, experimental studies and quantitative analysis using immunohistochemistry need to be conducted to better understand the molecular mechanisms of Cav-1 underlying our observations.

Application of the genetic variability of lung transplant recipients in the management and treatment of these patients could be a promising approach in the future. Genetic risk profiling might provide a tool for individualised risk stratification and for personalised immunosuppressive treatment after lung transplantation. Personalised immunosuppressive treatment might lead to better graft survival and less drug toxicity. The *CAV1 *genotype of SNP *rs3807989 *is associated with the development of BOS and therefore could be included in such a genetic risk profile.

## Conclusions

Our data demonstrate that the *CAV1 *SNP *rs3807989 *is associated with the development of BOS after lung transplantation and that Cav-1 serum levels are influenced by the composition of the coding gene. The risk allele associates with increased Cav-1 serum levels, and OB lesions might show increased Cav-1 expression. The mechanism through which increased Cav-1 expression contributes to the development of BOS needs to be explored further.

## Materials and methods

### Patients and clinical data

All lung transplant recipients who underwent transplantation in the Heart Lung Centre of the University Medical Centre in Utrecht, The Netherlands, in the period from July 2001 to November 2008 were asked to donate DNA and serum.

The diagnosis BOS was defined as a decline in forced expiratory volume in one second (FEV_1_) of greater than 20% from the baseline determined by average of two measurements made at least three weeks apart in the absence of known causes for acutely declining FEV_1_, such as acute rejection and infection [16]. Standard immunosuppressive therapy consisted of basiliximab (induction), tacrolimus, mycophenolate mofetil and prednisone for all patients. After approval by the local medical ethics committee, informed consent was obtained from each lung transplant recipient and healthy control, and DNA and serum were collected.

### Genotyping

Three haplotype-tagging SNPs for the *CAV1 *gene were selected using the Tagger programme (Broad Institute, Cambridge, MA, USA; http://www.broadinstitute.org/mpg/tagger/) for the genomic region of *CAV1 *± 2,500 bp on genome build 35. Preferential picking of SNPs was conducted under the pairwise tagging options, a minimum allele frequency setting of 25% and a high Illumina design score (Illumina; San Diego, CA, USA). The algorithm was set to select tags that would cover the Caucasian HapMap panel with an *r*^2 ^of 0.8 or more [42]. Furthermore, additional SNPs were selected on the basis of previously published data or presumed functionality. The following SNPs were genotyped: *rs12154695*, *rs10256914*, *rs3807989 *and *rs3807994*.

DNA was extracted from whole-blood samples, and SNP typing was conducted using a custom-made Illumina GoldenGate bead SNP assay (Illumina) in accordance with the manufacturer's recommendations. The characteristics of the lung transplant recipients and controls from whom DNA was taken are shown in Table 1. In three BOS^neg ^patients, the genotyping of the *CAV1 *SNPs failed. The control group comprised 422 healthy individuals who were not receiving any medical treatment at the time of analysis.

### Cav-1 serum levels in patient groups and healthy controls

Cav-1 serum levels were measured in different groups of lung transplant recipients to perform longitudinal analysis and to investigate whether Cav-1 can be used as a biomarker for BOS. For longitudinal analysis of Cav-1 serum levels, 10 BOS^pos ^patients were matched with 10 BOS^neg ^patients to reduce the influence of confounding factors. In this matched cohort of 10 BOS^pos ^patients and 10 BOS^neg ^patients, sequential serum samples (ranging from two to five samples per patient) were analysed. These patients were matched for several clinicodemographic variables to reduce the influence of confounding factors, including age (difference in age less than three years), gender, primary lung pathology, postoperative follow-up time (difference in postoperative follow-up time less than one year) and unilateral or bilateral transplantation (Table 4). Patients were matched on these five items with a median of 4.0 matching items (range, 2.0 to 5.0 items).

A quadrant-based sampling model was used to compare serum Cav-1 levels between the matched BOS^pos ^and BOS^neg ^patients at similar time points after lung transplantation and prior to BOS as described previously [43,44]. The time period from lung transplantation until the diagnosis of BOS was made varied in the cohort of BOS^pos ^patients with a mean of 19 months (Table 4). To investigate whether Cav-1 serum levels are useful as a biomarker for BOS, Cav-1 serum levels were measured at one moment in 33 BOS^neg ^patients who were not matched with a BOS^pos ^patient. The baseline characteristics of these three groups of lung transplant recipients are summarised in Table 4.

Cav-1 serum levels were measured in 60 healthy controls (Table 4). The minor allele frequencies of the four SNPs in this cohort were 37% (*rs12154695*), 30% (*rs10256914*), 50% (*rs3807989*) and 28% (*rs3807994*). To improve our understanding of the role of Cav-1 in pulmonary fibrosis, Cav-1 serum levels were measured in 25 patients with IPF at one time point (Table 4). These patients were diagnosed according to the current American Thoracic Society/European Respiratory Society guidelines [45].

### Protocol for serum Cav-1 assay

All serum samples were stored at -80°C until analysis. Serum Cav-1 was measured using the ELISA developed by Tahir *et al. *[22] with some minor modifications. Briefly, Nunc maxisorb microplate wells (Nalgene Nunc International/Thermo Scientific, Rochester, NY, USA) were coated overnight at 4°C with 100 μL of 0.25 μg/well polyclonal anti-Cav-1 antibody (BD Transduction Laboratories, San Diego, CA, USA) and blocked with Tris-buffered saline containing 1.5% BSA and 0.05% vol/vol Tween 20 (blocking buffer). All incubation was done at room temperature. To 50 μL of blocking buffer, 50 μL of serum samples, calibrators and controls were added. We used full-length Cav-1 recombinant protein as a calibrator (Abnova, Taipei City, Taiwan). After shaking, the plate was incubated for two hours. After washing, 100 μL of 0.1 μg/well monoclonal Cav-1 antibody (BD Transduction Laboratories) were incubated for 90 minutes, followed by 60 minutes of incubation with 100 μL of 0.13 μg/well polyclonal rabbit antimouse horseradish peroxidase (Dako, Glostrup, Denmark). After washing, 90 μL of 3,3',5,5'-tetramethylbenzidine substrate solution (BioLegend, San Diego, CA, USA) were added and the blue colour was allowed to develop for 20 minutes in the dark. The reaction was stopped by adding 50 μL of 2 N H_2_SO_4_, and the absorbance was read at 450 nm using a microplate reader (BioTek, Winooski, VT, USA).

### Immunohistochemistry of human lung tissue sections

Paraffin-embedded tissue was available from six BOS^pos ^patients (lung biopsy, autopsy or lung explant). Excess tissue of a lung donor and an area of normal lung tissue from a lobectomy specimen were used as healthy controls.

Serial cross-sections (4 μm) were deparaffinized and rehydrated, boiled in sodium citrate and blocked in 10% normal goat serum. The sections were incubated for one hour at room temperature with 1.25 μg/mL polyclonal rabbit antihuman Cav-1 antibody (BD Transduction Laboratories) as determined by titration, and analysed using the Novocastra PowerVision Poly-HRP Anti-Rabbit IHC Detection System (Leica Microsystems, Buffalo Grove, IL, USA). Staining was developed with 3,3'-diaminobenzidine substrate and counterstained with haematoxylin. Negative controls were obtained by avoiding the primary antibody.

### Statistical analysis

The statistical significance of the differences between groups was determined by using the χ^2 ^test and one-way analysis of variance. HWE and tests for association were calculated using the online programme available at http://ihg2.helmholtz-muenchen.de/cgi-bin/hw/hwa1.pl. The HWE cutoff for significant disequilibrium and subsequent exclusion from analysis was set at *P *= 0.05.

The significance threshold was set after accounting for multiple comparisons using a Bonferroni correction for the effective number of independent SNPs proposed by Li and Ji [46]. Owing to LD, the effective number of SNPs was three for *CAV1*, resulting in an adjusted significance threshold of 0.05/3 = 0.017. Therefore, *P *values were multiplied by three to adjust for multiple comparisons. Thus, *P *values ≤ 0.05 were considered statistically significant.

The LD structure of the polymorphisms was determined using Haploview 4.2 software [47]. Haplotypes were reconstructed using the PHASE software programme [48].

Cav-1 serum levels were not normally distributed and are expressed as medians with IQRs. To determine whether there was a trend in serial serum levels over time in a single subject, and to compare this trend between the two groups, a restricted maximum likelihood linear mixed model was used [49]. The Mann-Whitney *U *test was applied to comparisons between genotype and serum levels. Statistical analyses were performed using SPSS for Windows version 17.0 software (SPSS Inc, Chicago, IL, USA).

## Abbreviations

bp: base pair; BSA: bovine serum albumin; ELISA: enzyme-linked immunosorbent assay; SNP: single-nucleotide polymorphism.

## Competing interests

The authors declare that they have no competing interests.

## Authors' contributions

EAK participated in the design of the study, performed the statistical analysis and wrote the manuscript. CHMvM participated in the design of the study and the writing of the manuscript and performed the genotyping. KMK carried out the immunoassays. SMR carried out the immunohistochemistry and participated in writing the manuscript. HJTR participated in designing the study and writing the manuscript. JMKvE participated in performing research, collecting data, recruiting patients and writing the manuscript. EAvdG and DAvK participated in performing research, collecting data and recruiting patients. PZ performed the statistical analysis. JCG participated in performing research, designing the study, collecting data and recruiting patients. All authors read and approved the final manuscript.
